# Uterine collagen deposition fluctuates throughout the estrous cycle and provides a scaffold for gland visualization via the *SHG-casting* method

**DOI:** 10.1016/j.isci.2025.113989

**Published:** 2025-11-10

**Authors:** Audrey Savolainen, Huy Hoang Nguyen, Egor Panfilov, Veli-Pekka Ronkainen, Aleksei Tiulpin, Renata Prunskaite-Hyyryläinen

**Affiliations:** 1 Faculty of Biochemistry and Molecular Medicine, University of Oulu, 90220 Oulu, Finland; 2 Research Unit of Health Sciences and Technology, University of Oulu, 90220 Oulu, Finland; 3 Biocenter Oulu, University of Oulu, 90220 Oulu, Finland

**Keywords:** Imaging anatomy, Biological sciences, Physiology

## Abstract

Uterine extracellular matrix (ECM) and uterine glands are essential parts of the endometrium, with important roles in remodeling associated with the estrous cycle, fertility, and pregnancy. Using second harmonic generation (SHG) microscopy, enabling the label-free imaging of fibrillar collagen, we analyzed mouse endometrial tissue across estrous cycle phases. We observed quantifiable 2D and 3D changes in fibrillar collagen accumulation, especially around uterine glands in the estrus phase. We also validate that the 3D SHG fibrillar collagen scans can serve as a template to recreate 3D uterine gland morphology by a method we call *SHG-casting*, enabling simultaneous label-free studies of endometrial fibrillar collagen and glands. Our custom deep learning-based segmentation model enables rapid, accurate gland segmentation. *SHG-casting* reveals variable gland structures, including lobular morphology in estrus and involution in later phases. *SHG-casting* offers a powerful tool for the 3D imaging of epithelial structures supported by collagen-rich ECM, with broad research and diagnostic potential.

## Highlights

• Second harmonic generation (SHG) reveals uterine fibrillar collagen dynamics
• *SHG-casting* method enables label-free 3D uterine gland morphology reconstruction
• Deep learning model allows fast, accurate segmentation of uterine glands from scans
• *SHG-casting* has broad applications for collagen and epithelial structure studies

## Graphical abstract

**Figure:**
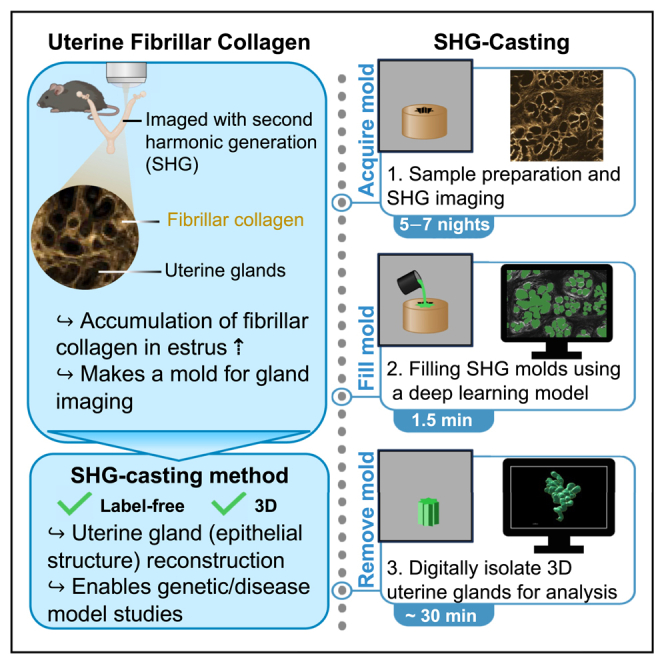

## Introduction

Infertility is an increasing concern for millions of couples globally. While assisted reproductive technologies, including *in vitro* fertilization, help many have a biological child, these technologies depend on a functional and receptive uterus. However, diagnostic markers and improved imaging are still needed, both in clinical and research laboratories, to determine when a uterus is most receptive and capacious for embryo implantation, pregnancy establishment, and maintenance to parturition, or bears fertility-ablating changes.^1^

The mammalian uterus is a remarkable organ, capable of tremendous adaptation to prepare for and support a pregnancy. It is comprised of three layers: the outermost perimetrium, the myometrium, and the inner lining, the endometrium.^2^ The endometrium is a heterogeneous tissue, primarily comprised of endometrial stromal fibroblasts, epithelial uterine glands (also referred to as endometrial glands), and luminal epithelium lining the lumen, all of which are surrounded and supported by extracellular matrix (ECM).^2^^,^^3^ The ECM is largely composed of fibrillar and non-fibrillar collagens, fibronectin, elastin, laminins, hyaluronidase, and proteoglycans.^4^ Though all endometrial cells deposit ECM, which interconnects them, endometrial fibroblasts are the primary source of stromal interstitial ECM.^3^^,^^4^ Regular endometrial remodeling is a physiological and necessary part of uterine health. Defective endometrial function is associated with uterine pathologies and fertility complications, including endometriosis, polycystic ovary syndrome, placental abnormalities, preterm births, miscarriage, and infertility.^5^

Mice are routinely used as a model organism to study human reproductive functions as they have an estrous cycle, similar to the menstrual cycle, which is regulated by hormonal fluctuations, namely estrogen (17β estradiol, E_2_) and progesterone (P_4_),^6^ and divided into four phases: proestrus, estrus, metestrus, and diestrus.^7^ The estrous cycle generally lasts about 4–5 days, during which significant remodeling in the endometrial stroma, ECM, and uterine glands occurs.^8^^,^^9^ During the estrus phase, E_2_ levels peak, and the endometrium is the thickest and filled with large, multi-lobular uterine glands.^8^^,^^9^^,^^10^ Following ovulation in the estrus phase and a decrease in E_2_, P_4_ levels rise, reaching their highest concentration in the diestrus phase, where the endometrium is the thinnest, with minimal uterine glands.^8^^,^^9^^,^^10^ The absence or dysfunction of the uterine glands has been shown to result in pregnancy loss and infertility.^11^^,^^12^^,^^13^ Importantly, the three-dimensional (3D) structure of uterine glands is genetically predetermined, and this 3D gland morphology correlates with their function, specifically impacting pregnancy outcomes.^13^

The ECM is an integral component of the endometrium, which historically was viewed as static,^14^ but has now attracted increasing attention as its profound role in morphogenesis and tissue integrity is better understood. Previous studies show that there are significant changes in endometrial collagen content across the estrous cycle.^8^^,^^15^ The ECM supports structure and cellular signaling through the reproductive cycles and pregnancy and is a proposed regulatory mechanism for adenogenesis, namely, uterine glands in the case of the uterus.^16^^,^^17^ Research in mammary gland branching, for example, suggests that collagen I plays a crucial role in branching morphogenesis.^18^ Mammary glands, such as uterine glands, also remodel significantly in relation to hormones, triggered by pregnancy, lactation, and weaning,^19^ and different fibrillar collagen expression patterns (for fibrillar collagens I, III, and V) have been associated with each remodeling event, suggesting that specific fibrillar collagens play key roles in mammary gland function and rearrangement.^19^ Interestingly, in a study using human mammary organoids, Buchmann and colleagues previously observed accumulations of collagens at the front of the mammary gland branches, which they described as “collagen cages.”^20^ They show that the collagen cage creates tension between the epithelial cells and the ECM, which induces the reorganization of the surrounding collagen, creating a stable guide for ductal branch elongation.^20^ The ECM and uterine glands are interdependent and integral parts of the functional uterus; however, they are rarely examined concurrently, leaving knowledge gaps in their co-development and potential applications in phenotyping, diagnostics, and treatment.

We recently demonstrated that second harmonic generation (SHG) microscopy is a powerful tool for detecting and visualizing fibrillar collagen accumulations around the implanted mouse embryo, comprising a distinct 3D collagenous decidual nest, which enables the measurement and evaluation of decidualization efficacy and pregnancy progression.^21^ Based on these discoveries and considering the previously reported uterine gland and ECM changes associated with the estrous cycle,^8^^,^^9^ and the observed collagen-cages encapsulating the mammary gland organoids^20^ we set out to apply SHG imaging to examine the 2D and 3D fibrillar collagen dynamics around uterine glands in the mouse uterus throughout the estrous cycle. This study reveals significant changes in fibrillar collagen deposition, particularly around uterine glands comprising distinct fibrillar collagen cages encapsulating glands. We also demonstrate that uterine glands generate a ‘negative impression’ in SHG scans (as they do not express fibrillar collagen), providing a clear template to recreate their 3D morphological structure without additional labeling. Based on these observations, we developed a method named *SHG-casting*, which can be applied as a label-free alternative to uterine gland morphology analysis. This method uses an SHG scan of the endometrium as a mold, and then, using manual segmentation or an automated deep learning-based semantic segmentation model, digitally “fills the mold” so that the 3D uterine gland “cast” can be analyzed. This method enables simultaneous uterine gland and fibrillar collagen studies, facilitating discoveries of their interdependency for downstream applications such as uterine gland characterization in genetically modified or disease mouse models. Moreover, the *SHG-casting* method serves as proof of principle for analysis in other research fields where fibrillar collagen-rich ECM surrounds an (e.g., epithelial) structure of interest, enabling its 3D reconstruction and correlative studies.

## Results

### Uterine stromal fibrillar collagen is deposited around the uterine glands in a distinct, estrous cycle-dependent manner

**Figure 1:**
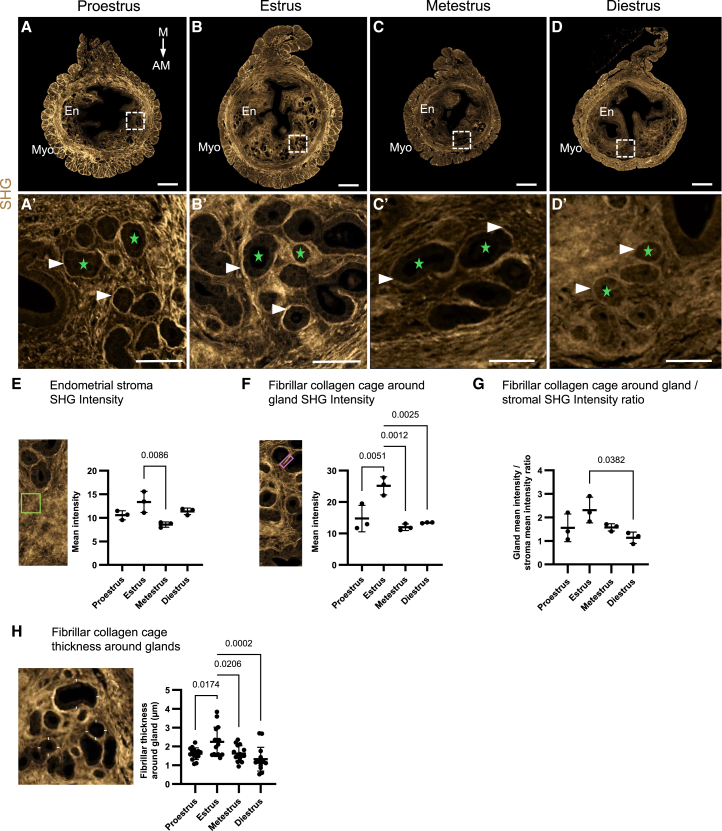
SHG intensity measurements in 2D reveal dynamic fluctuations in fibrillar collagen in the mouse uterus across the estrous cycle (A–D’) Representative paraffin-embedded uterine cross-section images depicting intrinsic SHG signal from (A) proestrus, (B) estrus, (C) metestrus, and (D) diestrus. The area of magnification is indicated with a white dashed box. A’–D’ Arrowheads point to the fibrillar collagen cage, and green stars mark the uterine gland place that appears like a “hole” in SHG scans. (E) Average endometrial stromal intensities from each estrous cycle stage. (F) Average SHG intensity of the fibrillar collagen cage, surrounding uterine glands, from each estrous cycle stage. (G) SHG signal ratio measurements coming from the fibrillar collagen cage surrounding uterine glands compared with uterine stromal SHG intensities. (H) Fibrillar collagen cage thickness measurements (obtained by the average of 4 measurements per gland, representative measurement locations shown with white lines on gland outline). (E–H) Graphs are accompanied by images showing representative examples from where the data was collected; green boxes indicate stromal region of interest (ROI), and the magenta box indicates fibrillar collagen cage ROI. AM, antimesometrial, En, endometrium, M, antimesometrial; Myo, myometrium, SHG, second harmonic generation. Statistical analyses in (E–H) were performed with one-way ANOVA followed by the Tukey post hoc test. *P* values below 0.05 are reported in the graph. (E–G) *n=*3 uteri/estrous cycle phase, and (H) *n* = 5 glands per uteri/estrous cycle stage. The results are shown as individual data points per uterine tissue section with mean ± s.d. The data points in E represent the average of three stromal intensity measurements per uterine tissue sections. The data points in F represent the average of five fibrillar collagen cage intensity measurements per uterine tissue section. The data points in G represent the average of five fibrillar collagen cage to stroma intensity measurements per uterine tissue section. The data points in H represent the average of four “compass measurements” per gland. Scale bars (A–D) 200 μm, (A’–D’) 50 μm.

It is already well validated that fibrillar collagen is specifically detected by SHG.^22^ We additionally demonstrated that SHG detects uterine endometrial fibrillar collagen in mice.^21^ To develop a method for the simultaneous imaging and analysis of uterine glands and fibrillar collagen, we first set out to map the fibrillar collagen accumulation in murine uteri through all estrous cycle stages, proestrus, estrus, metestrus, and diestrus, as determined by vaginal cytology (Figure S1). We scanned uniform thickness (5 μm) paraffin-embedded uterine cross-sections with SHG using the same image acquisition settings (Figures 1A–1D’). Note the strong SHG signal from the myometrium, which we reported earlier^21^ (Figures 1A–1D). Zoomed-in micrographs of the endometrium depict stromal fibrillar collagen outlining the uterine glands (glandular epithelium and lumen) and making them appear as black “holes” as they do not possess fibrillar collagens (Figures 1A’–1D’). We observed more intense SHG signals emitted from thin fibrillar collagen accumulations surrounding the epithelial uterine glands (Figures 1A’–1D’). We refer to them as fibrillar collagen cages due to their close resemblance to collagen accumulations described in mammary organoids by Buchmann et al*.*^20^ In endometrial stroma intensity measurements, estrus had a significantly higher intensity than metestrus (*p* = 0.0086, Figure 1E). The SHG intensity of the fibrillar collagen cage around the glands was significantly higher in estrus compared to the proestrus (*p* = 0.0051), metestrus (*p* = 0.0012), and diestrus (*p* = 0.0025) phases (Figure 1F). When we compared the SHG intensity depicting fibrillar collagen accumulation around the uterine glands to the surrounding stroma, we found that the SHG intensity of the fibrillar collagen cage was from 0.87 to 2.90-fold higher, depending on the estrous cycle stages and was significantly higher in the estrus phase fibrillar collagen cages compared to those in the diestrus phase (*p* = 0.0382, Figure 1G). Furthermore, we also observed that the fibrillar collagen cage was significantly thicker in the estrus phase compared to the proestrus (*p* = 0.0174), metestrus (*p* = 0.0206), and diestrus (*p* = 0.0002) phases (Figure 1H).

**Figure 2:**
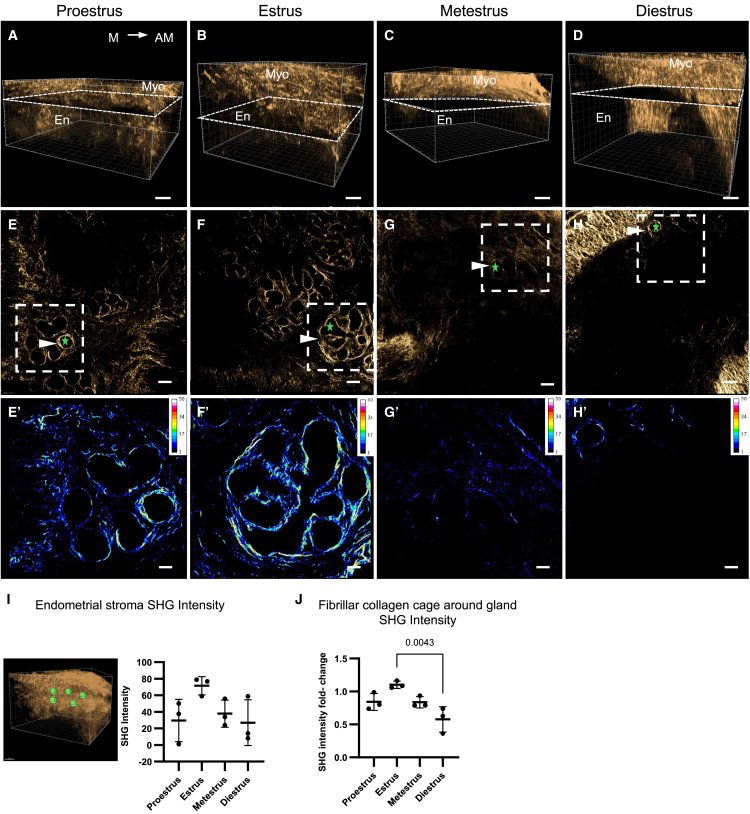
SHG intensity measurements in 3D scans reflect fluctuations in fibrillar collagen deposition in the mouse uterus across the estrous cycle (A–D) Representative 3D SHG scans of (A) proestrus, (B) estrus, (C) metestrus, and (D) diestrus uteri showing the myometrium and the endometrium. The location of the representative z stack slice (white dashed line) showing gland outlines is depicted later in discussion in (E–F). (E–H) Representative z stack images from 3D reconstruction showing variations in gland outlines in (E) proestrus, (F) estrus, (G) metestrus, and (H) diestrus scans. Arrowheads point to fibrillar collagen-gland cage, green star marks the uterine gland place that appears like a “hole” in SHG scans. (E’–H’) Fibrillar collagen cage enlarged ratio map showing the SHG intensity fold change of gland fibrillar collagen cage in relation to the endometrium in (E’) proestrus, (F’) estrus, (G’) metestrus, and (H’) diestrus scans. The entire image was divided by its mean gray value and is shown, scaled for changes from 1 to 50-fold. (I) Endometrial stroma SHG intensity measurements across the estrous cycle. Green cubes illustrate representative places from where SHG intensity measurements were taken. (J) SHG intensity fold-change measurements of differences in gland fibrillar collagen cages across the estrous cycle. AM, antimesometrial; En, endometrium; M, mesometrial; Myo; myometrium, SHG, second harmonic generation. Statistical analyses were performed with one-way ANOVA followed by the Tukey post hoc test. *P* values below 0.05 are reported in the graphs. The results are shown as individual data points with mean ± s.d., *n =* 3 uteri/estrous cycle phase. The data points in I represent the average of five ROI measurements per uterus. The data points in J represent one uterine gland z stack analyzed per uteri. Scale bars for (A–D) are 100 μm and for (E–H’) are 50 μm.

To further characterize endometrial fibrillar collagen dynamics, chemically cleared pieces of uterine horns from each phase of the estrous cycle were scanned with a multiphoton microscope using SHG viewed in 3D (Figures 2A–2D). To enable comparison and measurements, SHG imaging settings were kept the same for all 3D acquisitions. The myometrial fibrillar collagen generated a strong SHG signal, while in comparison, the endometrial signal was less intense and fluctuated through the estrous cycle, particularly in the fibrillar collagen cages surrounding uterine glands (Figures 2E–2H). To visualize the difference in SHG signal intensity in the fibrillar collagen cage encapsulating uterine glands in comparison to the surrounding stroma, we created representative fold-change ratio maps of the uterine gland signal compared to the mean intensity of the entire image from each phase of the estrous cycle (Figures 2E’–2H’). The data showed that the SHG signal from the fibrillar collagen cage was approximately 10-fold higher than the surrounding endometrial stroma, particularly in the proestrus and estrus phases (Figures 2E’–2H’). Endometrial stroma SHG intensity measurements were performed by taking an average of 5 small cubic ROIs placed in the endometrium, excluding lumen and glands (Figure 2I). No significant differences were observed, but the SHG intensity appeared highest in the estrus phase, consistent with the 2D SHG intensity measurements. To quantify differences in fibrillar collagen cage intensity in 3D, we created small image sub-stacks from each estrous cycle scan, encompassing uterine gland clusters (excluding myometrium and lumen), to analyze. Estrus fibrillar collagen cages had a significantly higher SHG intensity fold-change compared to diestrus fibrillar collagen cages (*p* = 0.0043, Figure 2J), in line with the measurements from the 2D uterine cross-sections (Figures 1F and 1G).

**Figure 3:**
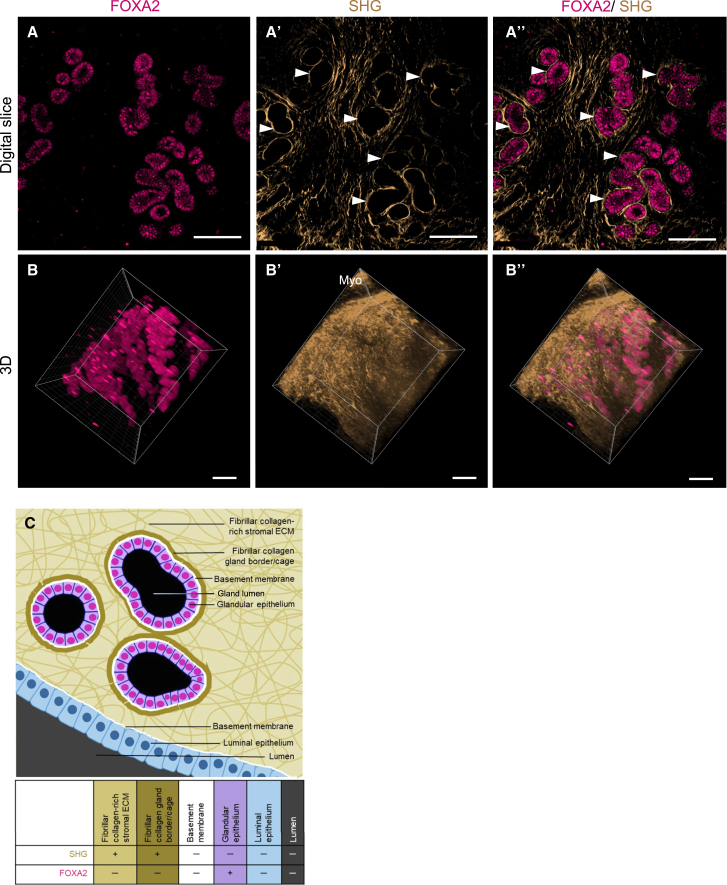
Second harmonic generation and immunostaining reveal endometrial fibrillar collagen cages surrounding uterine glands (A–B’’) Representative images from z stack image show (A) uterine gland-specific FOXA2 staining (magenta), (A’) SHG signal (gold) from fibrillar collagen cages around glands, and (A’’) merged FOXA2 and SHG. 3D reconstructions of whole-mount uterus (B) stained with FOXA2 antibody depicting uterine glands, (B’) SHG signal depicting fibrillar collagens, and (B’’) merged FOXA2 and SHG signals. (C) Fibrillar collagen-rich stromal ECM has a more intense SHG signal at the gland cage. A basement membrane (white) underlies the epithelium in the glands and lumen. Because it is mainly composed of collagen IV, a non-fibrillar collagen, it is SHG-negative. Glandular epithelium is FOXA2 (nuclear stain, magenta) positive but has no fibrillar collagen content. Luminal epithelium (light blue) and lumen (dark gray) do not contain fibrillar collagen or express FOXA2, so they are negative for all of the listed markers. Scale bars (A–B’') are 100 μm. *n* = 3 independently stained pieces from 1 uterus.

To confirm that the SHG signal we attributed to uterine gland fibrillar collagen cages surrounded the uterine glands, we performed whole-mount immunostaining of uterine tissue samples with a known uterine gland-specific nuclear marker, forkhead box A2 (FOXA2).^13^^,^^23^^,^^24^ We then imaged the mouse uterine samples with multiphoton microscopy, acquiring both FOXA2 immunofluorescence (Figure 3A) and SHG (Figure 3A’) signals. In 2D, z stack images, the fibrillar collagen is shown closely interweaving around the glands and in the endometrial stroma (Figure 3A’’), forming the fibrillar collagen gland cage. When reconstructed in 3D, uterine glands (Figure 3B) and the surrounding fibrillar collagen (Figure 3B’) show that the fibrillar collagen is closely distributed along the entire gland structures (Figure 3B’’). The schematic image in Figure 3C details the structures specifically recognized by the FOXA2 antibody and SHG in the uterine endometrium. The FOXA2 immunofluorescence staining is specific to glandular epithelium, whereas SHG is specific to fibrillar collagens sparsely distributed in the stroma and their denser accumulation at the uterine gland cage.

Taken together, the data show that endometrial fibrillar collagen deposition fluctuates throughout the estrous cycle and is distinct around the uterine glands, forming fibrillar collagen cages. Furthermore, the changes in fibrillar collagen deposition are measurable in 2D tissue cross-sections and 3D tissue scans by label-free SHG microscopy, being highest in estrus and lowest in diestrus, and consistently more intense around the uterine glands.

### *SHG-casting*: A method for the label-free reconstruction of uterine gland 3D morphology using fibrillar collagen as a mold

**Figure 4:**
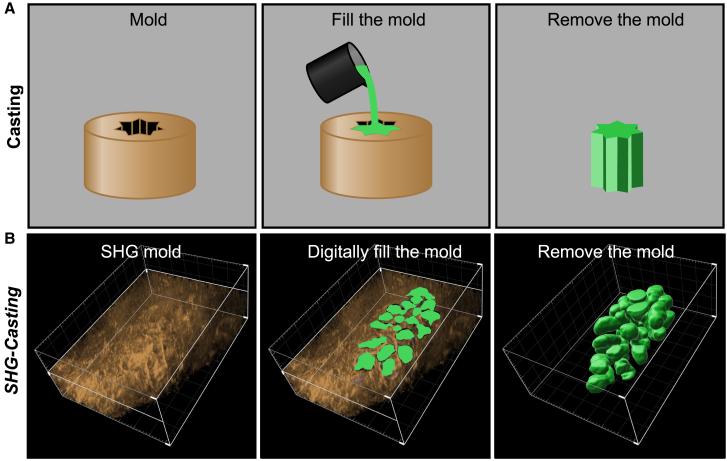
Schematic diagram illustrating the *SHG-casting* method (A) Conventional casting method where a mold, containing the hollowed form of the object of interest, is filled. When the mold is removed, the cast of the object of interest is revealed. (B) The *SHG-casting* method is shown, where the fibrillar collagen-rich extracellular matrix, detected by SHG, serves as a mold. The negative spaces in the extracellular matrix, the result of uterine glands not expressing fibrillar collagen, are digitally filled, resulting in the recreation of the 3D form of a uterine gland. SHG, second harmonic generation.

During our characterization of fibrillar collagen deposition in the uterus surrounding the uterine glands, we observed that no SHG signal came from the uterine glands themselves nor the uterine lumen (Figures 1A–1D’, 2A–2H, and 3A–3C). Moreover, the SHG signal consistently formed a fibrillar collagen cage around the glands, leaving a “negative impression” of the gland, particularly in the estrus phase (Figures 1B’, 2F, 2F’, and 3A’). We realized that these fibrillar collagen gland cages and the absence of SHG signal from within the gland could be used as a template for the 3D gland morphology reconstruction. We based this idea on the concept of casting: a method commonly used in manufacturing, where a mold containing a hollowed-out impression of a desired form is filled, and then the mold is removed, and the form of interest remains as a 3D cast (Figure 4A). We hypothesized that the SHG signal arising from fibrillar collagen in the stroma could serve as a mold, and the negative impression from the gland could be digitally filled, allowing for 3D, label-free gland reconstruction, which we named the *SHG-casting* method (Figure 4B).

**Figure 5:**
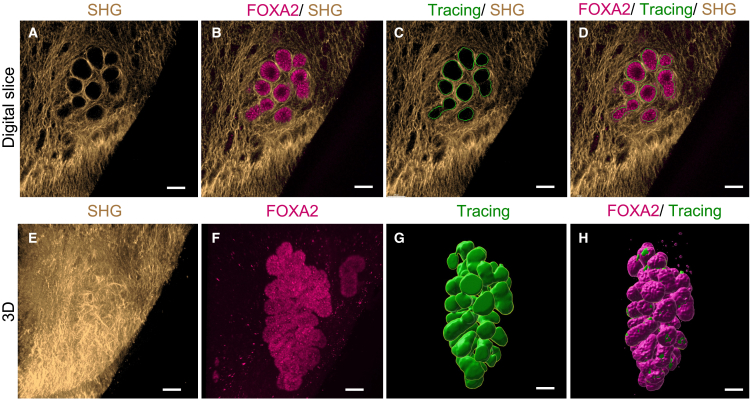
Fibrillar collagen detected by SHG provides a template for 3D gland reconstruction by the SHG-casting method (A–D) Representative 2D digital slice images of (A) fibrillar collagen detected by SHG (gold) forming a cage around the uterine gland and (B) uterine gland, identified by FOXA2 immunostaining (magenta). (C) Tracing of SHG outline (green) (D) aligns with FOXA2 staining (magenta). (E–H) 3D reconstruction of (E) SHG fibrillar collagen (gold), (F) FOXA2 staining of a uterine gland (magenta), (G) 3D reconstruction of gland tracing (green), and (H) merged uterine gland reconstructions of FOXA2 (magenta) and tracing (green). SHG; second harmonic generation. Scale bars are 50 μm. *n* = 2 manually traced scans.

Using images from a z stack containing signals both from SHG (Figure 5A) and FOXA2 immunofluorescence staining (Figure 5B), we tested our *SHG-casting* method hypothesis by manually tracing the SHG fibrillar collagen cage through the z stack of a uterine gland (Figures 5C and 5D). After tracing the fibrillar collagen cage throughout the SHG channel in the z stack (Figure 5E), we reconstituted the 3D uterine gland shape in immunostained FOXA2 (Figure 5F) and SHG scans (Figure 5G). When overlaid together, the FOXA2 3D gland structure is closely aligned with that of the manually traced SHG gland structure (Figure 5H), suggesting that the *SHG-casting* method could provide a label-free alternative to study uterine gland morphology and fibrillar collagen, with the possibility to complement the analysis with immunostaining. While we demonstrated it was possible to recreate the 3D uterine gland morphology using the SHG fibrillar collagen cage from around the gland as a template in this pilot study, we also found that the manual filling in of the gland outlines was tedious and time-consuming, prompting the development of an automated workflow.

### A deep learning-based segmentation model enables the automation of the *SHG-casting* method for the accurate reconstruction of the 3D uterine gland morphology

**Figure 6:**
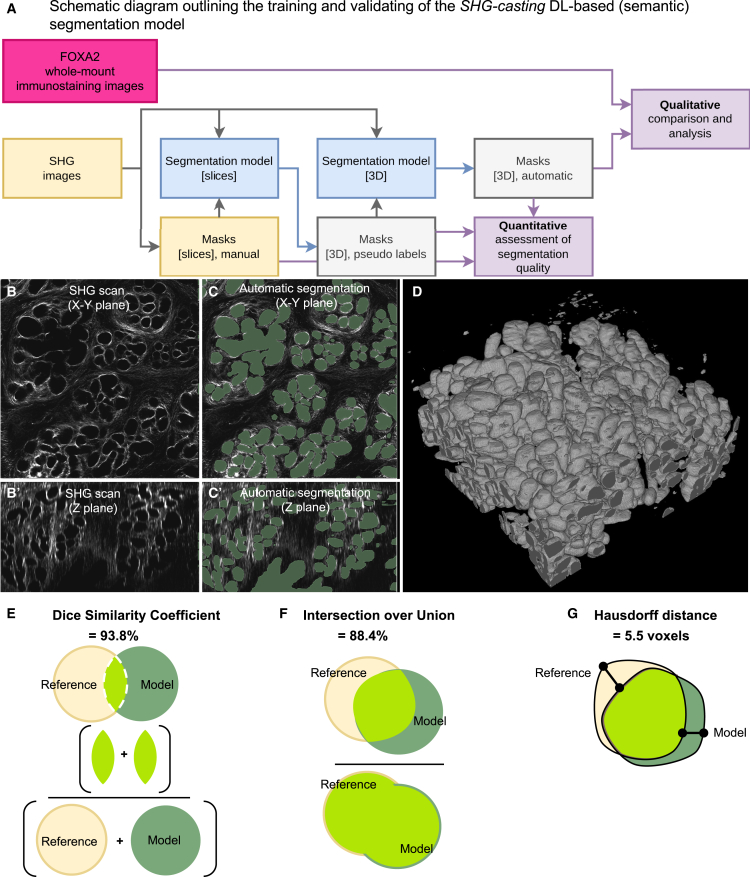
Deep learning-based segmentation model accurately recognizes and segments the fibrillar collagen cage (A) Schematic diagram shows the training and validation of machine learning trained model, featuring training to recognize the gland and lumen fibrillar collagen cages (SHG path), and to validate these segmentation and subsequent 3D reconstruction against that of FOXA2 uterine gland-specific whole-mount immunostaining (immunostaining path). (B and C) Representative images of 2D slices of (B, B’) SHG scan showing the negative imprints of uterine glands surrounded by fibrillar collagen cages in the x–y (top, B) and z (bottom, B’) planes, and the (C, C’) automatic segmentation of uterine gland imprints in the same regions in the x–y (top, C) and z (bottom, C’) planes. (D) The 3D model of uterine gland morphology resulting from the automatic segmentation of the complete SHG scan. (E) The Dice similarity coefficient (DSC), which compares the overlap of the reference 3D reconstruction to the *SHG-casting* segmentation model 3D reconstruction, is calculated by adding the overlap between the two methods together (lime green), divided by the total volume of the reference 3D model (yellow) with the volume from the *SHG-casting* segmentation model (green). The DSC is 93.8%. (F) Intersection over union (IoU) is a segmentation metric that measures the area overlap (intersection, bright green) between the *SHG-casting* segmentation model (green) and that of the reference (yellow), divided by the total area of both the model and reference (green) combined (union, bright green). The IoU score was 88.4%. (G) Hausdorff distance (HD) is a measure that assesses the worst case difference in distance between two sets of points in the model (green) and the reference (yellow). The HD is 5.5 voxels. Representative images to describe DSC, IoU, and HD were based on images from Pálsson et al.^25^ and Huynh.^26^

To improve the usability of the *SHG-casting* method, we developed a deep learning-based (DL-based, also referred to as “automated”) model to segment the SHG gland fibrillar collagen cages from SHG scan z-stacks for 3D gland reconstruction. Since this DL-based segmentation model was part of novel method development, we divided the work into two phases: 1) the model needed to learn to correctly segment the negative gland impression in the stromal fibrillar collagen, which appeared as empty holes in the fibrillar collagen-rich stroma, and 2) the SHG gland segmentation then needed to be validated and compared against the whole-mount immunostaining of uterine glands (the current segmentation method, Figure 6A). For the training, we used eight uterine gland scans: four scans were used for training, two were used for validation, and two were used for testing. Training scans were used to teach the model to correctly identify the fibrillar collagen gland cage. The validation scans were used for model snapshot selection. The performance of the model was assessed by how well it could segment the fibrillar collagen cages in the testing scans and how closely these segmentations matched uterine gland whole-mount immunostaining.

First, we trained the DL-based segmentation model to recognize the fibrillar collagen gland cages in the z stack slices (Figure 6A, yellow box, “SHG images”) using a supervised learning approach (Figure 6A, blue box, “segmentation model (slices)”). Because the uterine glands and the lumen both appear as negative space with an SHG signal outline, while the surrounding fibrillar collagen stroma gives the signal, the model was trained to segment both gland and lumen negative space up to the SHG outline, while ignoring the stromal SHG signal. As the SHG signal around the glands was not always continuous, or in cases where part of the cage was excluded from the imaging field, resulting in an incomplete outline, manual tracing was required to improve the segmentation (similar to that used in the pilot study, Figure 5C). We manually annotated every 15^th^ slice from eight scans, for a total of 203 annotated slices (Figure 6A, yellow box, “Masks (slices) manual”). The manual annotations were used to train the 2D segmentation model, which was subsequently used to generate pseudo labels for the complete dataset (Figure 6A, gray box, “Masks (3D) pseudo labels”). Next, to ensure higher border consistency in automatic segmentations, a 3D segmentation model was developed based on the pseudo labels (Figure 6A, blue box, “Segmentation model (3D)”). The 3D segmentation model was then applied to the entire stack to automatically segment the scans in 3D (Figure 6A, gray box, “Masks (3D), automatic). The performance of 2D and 3D models was assessed with respect to manual slice-wise segmentations and pseudo labels, respectively (Figure 6A, purple box, “Quantitative assessment of segmentation quality”). After the development, the 3D model was used to produce the final segmentations for the complete dataset. A representative example thereof is shown in Figures 6B–6D.

The performance of automatic segmentation was assessed based on the established medical image segmentation metrics: Dice similarity coefficient (DSC), intersection-over-union (IoU), Hausdorff distance, and sensitivity and specificity. The DSC, also known as the overlap index, measures the ratio between two times the overlapping volume and the sum of the ground truth volume and the predicted volume (Figure 6E).^27^^,^^28^ IoU, also known as the Jaccard index or Jaccard similarity coefficient, calculates the area of intersection between the reference manual annotations and model areas, divided by the total area (or the union, Figure 6F).^27^ The Hausdorff distance represents the worst-case difference between the reference and the predicted segmentations (Figure 6G).^27^ The 2D slice model has shown a DSC of 94.2%, an IoU score of 89.4%, a Hausdorff distance of 22.3 pixels, a sensitivity of 94.4%, and a specificity of 97.3%. For the 3D model, the DSC was 93.8%, the IoU was 88.4%, the Hausdorff distance was 5.5 voxels, and the sensitivity and specificity were 97.0% and 96.7%, respectively. Overall, these metrics indicate that the 2D and 3D models can accurately segment the negative space in the SHG scans delineated by fibrillar collagen cages and the lumen.

**Figure 7:**
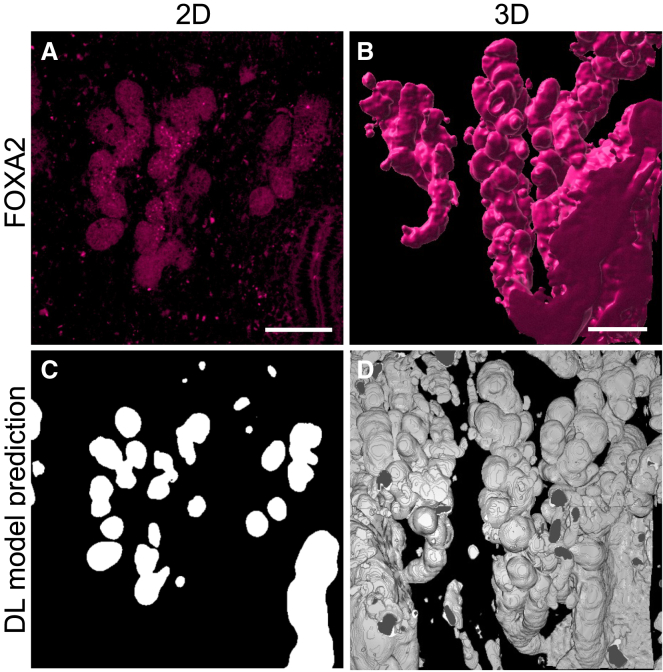
Comparison between uterine gland structure derived from FOXA2 immunostaining and SHG-casting method in combination with deep learning (DL) based (semantic) segmentation model (A) 2D digital slice of FOXA2 uterine gland staining and (B) 3D reconstruction of staining. (C) 2D segmentation map from *SHG-casting* DL-based segmentation model from the same digital slice shown in (A), followed by 3D reconstruction in (D). Scale bar for (A) is 50 μm and 100 μm in (B).

Once the automatic segmentations for the gland and lumen in the SHG z-stacks were generated, we then qualitatively compared the segmentations from the model (referred to as “predicted”) to FOXA2 whole-mount immunostaining (specific to uterine glands), which was imaged in addition to SHG in the same scan (Figure 6A, purple box, “Qualitative comparison and analysis”). This enabled us to directly compare the uterine gland structures across the methods. The FOXA2 antibody-stained uterine glands were considered “ground truth” in this phase of analysis. The FOXA2 immunostaining showed long, multi-lobular glands originating from individual stalks (Figures 7A and 7B). The model prediction also showed a very similar structure, where the glands protruded from a single stalk, with an elongated and multi-lobular structure (Figures 7C and 7D). It is important to note that because the model considers all negative space in the ECM, the lumen and vasculature are also included in the predicted structure. From the model prediction, the connection of the gland to the lumen is visible, and the absence of FOXA2 expression in the lumen enabled the segmentation result analysis only for uterine glands. Taken together, qualitative analysis demonstrates a high level of similarity in uterine gland structure obtained by the FOXA2 whole-mount immunostaining and automated *SHG-casting* method in the DL-based segmentation model.

### *SHG-casting* method enables label-free 3D single uterine gland visualization across the estrous cycle scans

**Figure 8:**
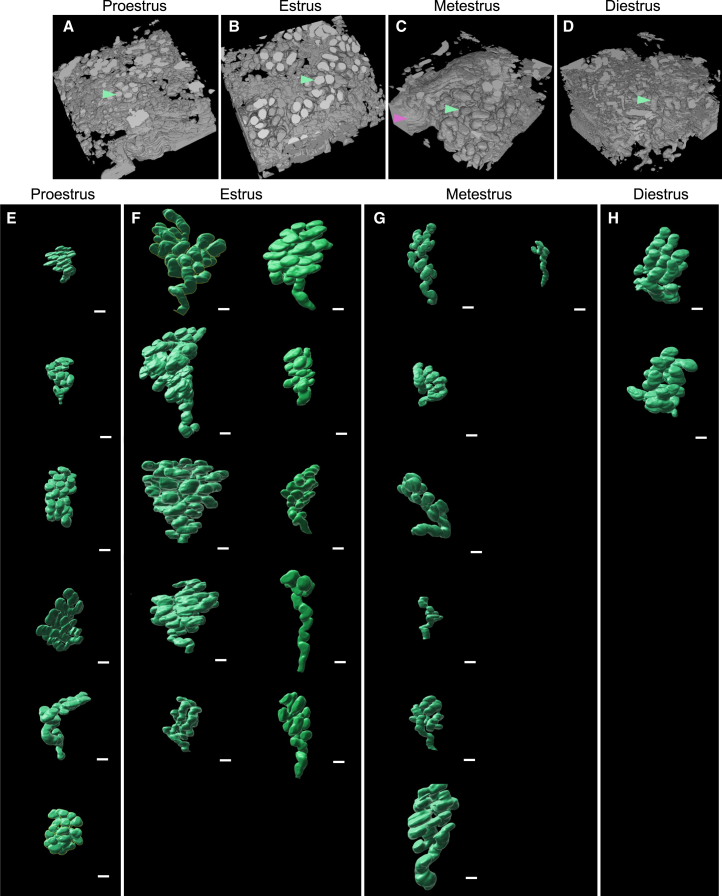
*SHG-casting* segmentation model output overview, followed by single uterine gland reconstruction (A–D) Representative *SHG-casting* segmentation models showing reconstructions of estrous cycle scans from (A) proestrus, (B) estrus, (C) metestrus, and (D) diestrus. The models have been cropped to emphasize the uterine glands and exclude the myometrium. Green arrows in A–D point to gland tips, and the pink arrow in (C) to segmented lumen. (E–H) *SHG-casting* segmentation method enables single uterine gland reconstruction. The array of single uterine glands reconstructed from the *SHG-casting* method with the DL-segmentation and manually separated from the lumen (E) proestrus, (F) estrus, (G) metestrus, and (H) diestrus phases. DL, deep learning; SHG, second harmonic generation. Each reconstruction is from a separate uterus scan. *n =* 3 uteri/estrous cycle phase. Scale bars in (E–H) are 50 μm.

After training and validating the DL-based segmentation model, we applied the *SHG-casting* method to the 3D SHG scans we collected for SHG intensity analysis (Figures 6A–6D, 8A–8D, and S2A–S2L). All scans could be segmented (Figures 8A–8D and S2A–S2L). However, there were variations in the quality of the segmentation, a direct result of the differences in fibrillar collagen gland cage intensity between the estrous cycle stages, as all 3D scans were acquired with the same settings for comparison purposes. In the estrus stage, where the SHG signal around the glands was the strongest, we also had the best segmentation results, with dense, lobular gland structures observed (Figures 8B and S2D–S2F). In proestrus and metestrus, multi-lobular gland structures were also present; however, the reconstructed glands were less consistent, with more breaks in the segmentations (Figures 8A, 8C, S2A–S2C, and S2G–S2I). The diestrus scan reconstructions were the most fragmented, in line with the observed lowest fibrillar collagen accumulation along the gland surrounding collagen cage, leading to low contrast between the stroma and the glandular epithelium when using similar acquisition parameters for all specimens (Figures 1F, 1G, 2I, 8D, and S2J–S2L).

To further test how the automated *SHG-casting* method could be used for gland morphology analysis, segmentation maps of the SHG scans (Figures 8A–8D and S2A–S2L) were analyzed using Imaris 3D image analysis software to assess whether it would be possible to isolate individual uterine glands. The analyses enabled the successful isolation of single uterine glands from proestrus (six glands), estrus (ten glands), metestrus (seven glands), and diestrus (two glands) SHG uterine scans applying the automated *SHG-casting* method (Figures 8E–8H and S2M). We observed coiled glands with multiple lobes in all the isolated uterine glands, but their morphology varied by estrous cycle stage (Figures 8E–8H). Glands in estrus were observed to be larger, both in length and width, with large lobes (Figure 8F compared to 8E). In metestrus, the glands appeared overall narrower (Figure 8G). The diestrus glands appeared lobular, but their segmentation was the least effective, attributed to the lowest fibrillar collagen cage accumulation (Figure 8H). As a note, the lumen and vasculature, also segmented in the automated model, were manually removed to visualize the single uterine glands.

**Figure 9:**
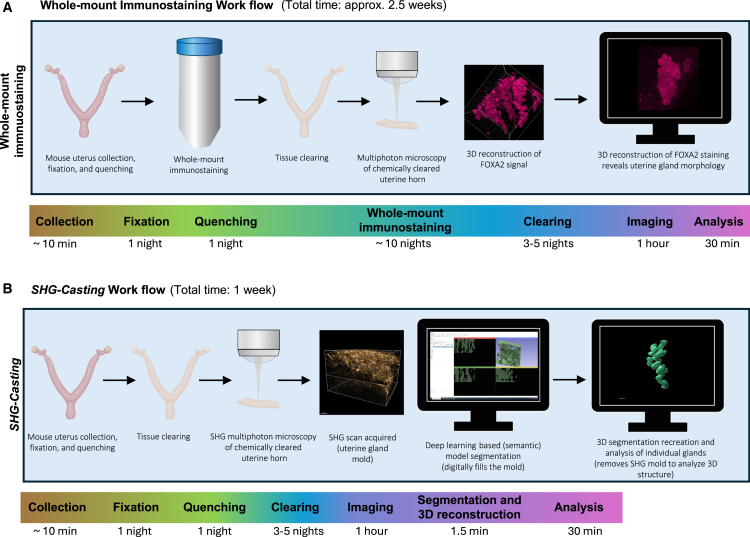
Comparison of workflow and time required for uterine gland 3D morphology reconstruction by whole-mount immunostaining and the automated *SHG-casting* method The estimated time for each step includes both hands-on work and passive waiting gaps. Scanning and analysis times are for one sample. (A) depicts the workflow of uterine gland 3D morphology reconstruction by whole-mount immunostaining, and (B) by the automated *SHG-casting* method. The initial steps involving specimen collection, fixation, and quenching of background signal are identical in both methods (A and B). The most significant time-related deviation comes at the immunostaining stage, taking up to 10 nights (A). Meanwhile, specimens processed by the *SHG-casting* method can already be chemically cleared for imaging (B). Critical to note that the SHG signal coming from fibrillar collagens is not sensitive to bleaching, enabling repetitive re-imaging of the specimens (B), whereas imaging a fluorophore associated with an antibody is bleaching the signal permanently (A). SHG; second harmonic generation. Uterus graphic is from BioRender: Savolainen, A. (2025) https://BioRender.com/jie1q29.

All in all, this proof-of-principle study showcases that it is possible to reconstruct single uterine gland 3D morphology by the presented manual and automated *SHG-casting* method, enabling future, deeper uterine gland analyses as such or in combination with fibrillar collagen and other markers. Furthermore, it provides validated means to shorten the time and resources needed to perform such analysis, as currently, the workflow to analyze uterine gland morphology using whole-mount immunostaining takes about 2.5 weeks to complete (from sample collection through analysis, including approximately 10 nights for whole-mount immunostaining (Figure 9A).^13^^,^^23^^,^^29^^,^^30^ Conversely, the *SHG-casting* workflow (from sample collection through analysis) takes about 1 week, as gland segmentation and 3D reconstruction for a single scan only takes about 1.5 min (Figure 9B). Therefore, automated *SHG-casting* has the potential to save up to 1.5 weeks in sample preparation time and costs for gland morphology analysis (Figures 9A and 9B).

## Discussion

In this study, we first characterize changes in the endometrial fibrillar collagen accumulation across the estrous cycle in mice in 3D using SHG microscopy. Secondly, we demonstrate that endometrial fibrillar collagen, detected by SHG, has the capability of serving as a template for a new method, *SHG-casting*, to extract 3D morphology from the impressions left in the ECM. Together, the findings in this article present novel insights into endometrial dynamics and gland morphology across the estrous cycle, which have exciting prospects for a better understanding of gland function and the role of fibrillar collagen in endometrial remodeling.

As SHG can detect endometrial fibrillar collagen,^21^^,^^22^ our studies add to the previous findings of changes in collagen content in relation to the estrous cycle stage.^8^ The study by Wood et al. investigated collagen I expression levels in mouse uterine tissue sections by quantifying immunostaining intensity and showed that intensity was strongest in estrus and lowest in the metestrus stage.^8^ We have very similar results to the collagen I changes observed by Wood et al. when analyzing the SHG intensity in the endometrial stroma. In 2D paraffin-embedded sections, our SHG intensity measurement in the stroma was significantly higher in the estrus phase compared to that in the metestrus phase. In our 3D stromal SHG intensity measurements, we again observed the highest intensity in the estrus phase, though not significantly higher than the other phases, possibly due to variability in the tissue depth, clearing, or sample size. Though considering the significantly higher expression of the stromal SHG intensity in the 2D measurements, and the high SHG intensity in the 3D, it is highly indicative that estrus-specific changes are occurring in endometrial stromal fibrillar collagen during the estrous cycle. Earlier studies showed that collagen I is the most abundant uterine collagen in nulliparous mice.^31^ Uteri of nulliparous mice also contained detectable levels of collagen III, whereas collagen V was not detected at all by SDS-PAGE.^31^ While SHG imaging cannot distinguish between specific fibrillar collagens, the results of Teodoro and colleagues^31^ and the collagen I staining intensity observed by Wood and colleagues^8^ throughout the estrous cycle, which resembled the fibrillar collagen accumulation intensity peaks depicted by SHG in this study, suggest that collagen I is likely the main source for the SHG signal in the non-pregnant uterus. However, it will be important to determine the ratios of collagen I and collagen III and if they have a similar accumulation pattern during the estrous cycle.

The ECM has long been credited as a driving factor in branching morphogenesis for epithelial structures, with involvement in lung, kidney, mammary gland, and salivary gland development.^32^ The role of the extracellular matrix has been previously reported to affect gland branching and was summarized by Khan and colleagues in 2022; however, no studies related to uterine glands were included^33^ providing support that the role of ECM in uterine gland branching is a poorly studied field. Similar to mammary glands, uterine glands develop postnatally and are known to remodel and dramatically change in morphology in relation to hormonal changes, pregnancy, lactation, and weaning.^34^^,^^35^^,^^36^ Our 3D analysis revealed fibrillar collagen cages surrounding the uterine glands, which appear to be characteristic of the estrus phase, as they are less pronounced or nearly absent in other phases. By imaging mammary glands in relation to collagen I, Brownfield and colleagues observed that collagen fibers were consistently found ahead of the epithelium and that the collagen I fibers were significantly co-oriented with the mammary gland bud end directions, suggesting that the collagen I fibers serve as a guide for the epithelial gland branch growth.^37^ Therefore, it is highly plausible that endometrial ECM, particularly collagen I, plays a significant role in uterine gland development and consequently gland function. Our research shows that there is an increased deposition of fibrillar collagen around the glands in the estrus phase, likely akin to the previously reported collagen cages,^20^ which may play a role in estrous cycle-associated morphological changes. We also demonstrate that SHG microscopy is a powerful imaging modality for going forward to learn more about stromal cell effects on uterine gland morphology in relation to the estrous cycle and pregnancy.

Based on our observation of fibrillar collagen gland cages around uterine glands, we developed a method called *SHG-casting*, as the process closely resembles the casting process in manufacturing to mass-produce a 3D object: a mold is created, the mold is filled, and the mold is removed, leaving the cast behind. While casting is a conventional method in manufacturing, in our method, we use the SHG signal derived from stromal fibrillar collagen cages as a digital mold, since the fibrillar collagen-rich ECM closely surrounds epithelial structures, such as uterine glands, in the tissue. Using a digital filling process (automatic segmentation of the uterine gland impression), the SHG mold is “filled”, leaving behind a 3D “cast” of the structure (in this case, the uterine glands) that was supported by the fibrillar collagen-rich ECM. The results of our pilot study, in which we manually filled in the SHG mold by tracing the gland perimeters throughout the z stack, convincingly demonstrated that indeed, a label-free fibrillar collagen SHG mold is suitable for recreating the uterine gland 3D morphology. Furthermore, since manual SHG mold filling (gland tracing) was tedious, we further optimized the *SHG-casting* method by developing a segmentation model that could automatically segment the uterine glands that appear such as “holes” in the SHG signal. Now, the *SHG-casting* method with automated segmentation increases the speed of analysis, as the process can bypass whole-mount immunostaining, saving about 1.5 weeks of tissue processing time and protocol optimization, and money spent on antibodies. The 3D *SHG-casting* segmentation model performed well with a DSC of 93.8%, IoU of 88.4%, and HD of 5.5 voxels. While DSC and IoU are both measures that assess the similarity in segmentation, the main difference between DCS and IoU is that over- and under-segmentation are penalized more in IoU.^27^ Aside from differences in how DCS and IoU are calculated, this is a likely reason for the lower IoU score compared to DSC. The comparison of uterine gland 3D structures derived from whole-mount immunostaining and those generated using the *SHG-casting* method revealed high similarity between the glands visualized by the different methods, enabling label-free visualization of uterine glands.

As the 3D analysis presented herein showed that fibrillar collagen deposition varies across the estrous cycle, reflected in the recorded SHG intensity changes, it is important to consider the impact that the estrous cycle stage has on the applicability of the *SHG-casting* method for uterine glands. We show that SHG intensity peaks in the estrus cycle, resulting in the best/sharpest fibrillar collagen-gland cages for the model to recognize. The scans from the diestrus stage, acquired with the same settings, showed more fragmented fibrillar collagen gland cages, causing challenges for the automated segmentation model to detect complete uterine gland cage SHG outlines and resulting in fewer uterine glands for analysis. This physiological fluctuation in fibrillar collagen was reflected in the segmentation-model’s ability to fragment and recreate the 3D gland structure, especially in diestrus when SHG intensity was lowest at the fibrillar collagen gland cage. This limitation could be overcome by using additional imaging methods and post-processing (i.e., Fluorescence Lifetime Imaging Microscopy (FLIM)) to remove background to enhance the outline around the glands. For this study, all our scans were acquired with the same settings deliberately for comparison purposes, illustrating that the scanning parameters would benefit from optimization based on the estrous cycle stage and study goals.

To the best of our knowledge, this is the first report characterizing endometrial fibrillar collagen fluctuations in relation to the estrous cycle with label-free SHG in 3D and the first proposed application of using SHG scans of the surrounding fibrillar collagen as a mold to reconstruct 3D epithelial structures (uterine glands) nestled within the ECM-rich stroma. This study demonstrates a proof-of-principle of the *SHG-casting* method to accurately reconstruct uterine gland morphology, potentially saving time and funds typically spent on immunostaining. Furthermore, this study serves to showcase the power of SHG imaging for uterine studies, which will hopefully inspire additional studies where both uterine morphology and fibrillar collagen localization analysis can be combined, maximizing data output from a single specimen. As fibrillar collagens have been shown to have key roles in mammary gland morphogenesis and function,^18^^,^^19^ endometrial extracellular collagens likely have profound implications for uterine gland morphogenesis, gland function, and fertility. The *SHG*-*casting* method simultaneously provides novel insights into both gland morphology and the surrounding fibrillar collagens, particularly useful in mouse models with genetic modifications or fertility defects. When looking to future applications for the *SHG*-*casting* method, human endometrial biopsies, containing uterine glands and surrounding fibrillar collagen-rich ECM, could be chemically cleared and scanned using the *SHG-casting* method. Furthermore, we foresee that the *SHG-casting* method has many applications for studies of other tissue types, as stromal fibrillar collagen-rich ECM surrounding various epithelial structures is abundant in the body and, for example, involved in renal, mammary, and salivary gland morphogenesis, and vasculature formation.^18^^,^^38^^,^^39^^,^^40^^,^^41^ Moreover, this study demonstrates that SHG signal analysis is a sensitive tool for identifying changes in ECM component accumulation, namely fibrillar collagens, which can be of high importance in the early diagnostics and monitoring of metabolic and fibrotic diseases. As technology and machine learning continue to improve, *SHG-casting* has immense potential for research and diagnostic use for rapid, label-free 3D morphology analysis.

### Limitations of the study

Here, we assessed changes in the endometrial fibrillar collagen using SHG and validated the *SHG-casting* method for label-free, 3D reconstruction of endometrial glands from the SHG fibrillar collagen scans. There are several limitations to our study, though. Firstly, due to the need for chemical tissue clearance prior to imaging, this method is currently only suitable for *ex vivo* specimen imaging. However, developments in imaging technologies, such as an SHG scanning endomicroscope, show that there could be future *in vivo* applications and exciting opportunities for real-time analysis of changes in uterine fibrillar collagen.^42^^,^^43^^,^^44^ Secondly, there are differences between the visual data acquired from FOXA2 staining and SHG. FOXA2 stains glandular nuclei, whereas the SHG signal comes from stromal fibrillar collagen gland cage, encapsulating glandular basement membrane and glandular cells with the cytoplasm. Because of these differences, we have qualitative comparisons of the *SHG-casting* automated model performance compared to whole-mount immunostaining for glands. This will also be important to consider in cases when glandular volumes would be compared using these two different gland visualization methods. This study is a qualitative proof of principle, and future studies to assess volumes, morphology, and other quantitative metrics would provide more insights and applications for this methodology. Lastly, our study shows that there are noticeable differences in the quality of gland segmentations across the estrous cycle scans due to biological differences in fibrillar collagen deposition around glands (highest in estrus and lowest in diestrus stages). As lower fibrillar collagen levels in diestrus result in poorer SHG signals, namely, the fibrillar collagen is not continually running around the gland rim, the *SHG-casting* results using automated filling were more fragmented. Therefore, there is little use of signal enhancement by increasing laser power, gain, pixel density, or oversampling, as this also leads to a poor noise to signal ratio. Our study shows that for label-free 3D gland reconstruction, it is best to choose estrus stage specimens. Should diestrus gland morphology be of specific interest, additional image optimization (previously mentioned as additional imaging methods and post-processing, (i.e., FLIM), could be useful for overcoming this limitation. Overall, further technological advancements, additional samples, imaging optimization, and quantitative metrics will enhance this study and broaden its application.

## Resource availability

### Lead contact

Requests for further information and resources should be directed to and will be taken care by the lead contact, Renata Prunskaite-Hyyryläinen (renata.prunskaite@oulu.fi).

### Materials availability

This study did not generate new unique reagents.

### Data and code availability

• Any additional information required to reanalyze the data reported in this article is available from the lead contact upon request.
• All original code has been deposited on GitHub at https://github.com/imedslab/mice_uterine_gland_segmentation and is publicly available as of the date of publication.

## Acknowledgments

We acknowledge the Oulu Laboratory Animal Center Research Infrastructure, University of Oulu, as well as Biocenter Oulu Light Microscopy and Transgenic and Tissue Phenotyping Core Facilities, supported by the University of Oulu and 10.13039/501100013840Biocenter Oulu Finland, for providing expert services and resources. We acknowledge Maliha Mehnaz Enamul for her assistance in performing measurements for Figure 1H. We thank Dr. Emmi Kapiainen for her valuable feedback on the article.

## Author contributions

Conceptualization, A.S. and R.P.-H.; methodology, A.S., H.H.N., E.P., A.T., and R.P.-H.; investigation, A.S., V-P. R., and R.P.-H.; software, H.H.N., and E.P.; writing – original draft, A.S., and R.P.-H.; visualization, A.S. and E.P.; writing – review and editing, all authors were given the opportunity to revise and comment on the article; funding acquisition, A.S., A.T., and R.P.-H.; resources, A.T. and R.P.-H.; supervision, A.T. and R.P.-H.

## Declaration of interests

The authors do not have any competing or financial interests to declare.

## STAR★Methods

### Key resources table

**Table:** 

REAGENT or RESOURCE	SOURCE	IDENTIFIER
**Antibodies**		

Rabbit monoclonal anti-FOXA2	Abcam	Ab108422, EPR4466; RRID:AB_11157157
Anti-Rabbit Alexa Fluor A594	Jackson ImmunoResearch	711-585-152, Lot # 145043; RRID:AB_2340621

**Chemicals, peptides, and recombinant proteins**		

*ScaleCUBIC-1*	Susaki et al.^45^	–

**Experimental models: Organisms/strains**		

C57BL/6NCrl mice	Charles River Laboratories, France	RRID:IMSR_CRL:027

**Software and algorithms**		

Imaris 9.8.2	Bitplane, Concord MA, USA	RRID:SCR_007370 http://www.bitplane.com/imaris/imari
3D Slicer 5.6.2	–	RRID:SCR_005619 https://www.slicer.org
Fiji (imageJ)	Schindelin et al.^46^	RRID:SCR_002285 http://fiji.sc/
GraphPad Prism 10.1.2	–	RRID:SCR_002798 https://www.graphpad.com/features
OriginPro 2024	OriginLab	https://www.originlab.com

**Other**		

Deep learning-based segmentation model	This paper	GitHub repository https://github.com/imedslab/mice_uterine_gland_segmentation

### Experimental model and study participant details

This study included 16 female C57BL/6NCrl mice (8 to 21 weeks old), born in the Oulu Laboratory Animal Centre (OULAC), University of Oulu, Finland. Mice were group-housed in conventional unit facilities in open cages (36.7 x 20.7 x 14 cm or 42.5 x 26.6 x 15 cm) with Aspen shavings for nesting material (PM90L/R, Tapvei, Estonia). Mice also had a cardboard hut (Mouse-house II) and a cardboard tunnel for enrichment. The room temperature was 21 ± 2°C, relative humidity 40% and illumination was 12-hour light/ 12-hour dark cycles. Mice received food pellets (SAFE® 150 Safe Custom Diets) and water *ad libitum.*

Animal handling was conducted in accordance with OULAC, University of Oulu, Oulu, Finland and Finnish institutional animal care policies, which fully meet the requirements of the European Union Directive 2010/63/EU and European Convention for the protection of vertebrate animals used for experimental and other scientific purposes (ETS No. 123, appendix A).

### Method details

#### Estrous cycle monitoring

Mouse estrous cycles were followed by checking vaginal cytology each morning at the same time. Briefly, a small amount (approximately 100 μL) of sterile 1× phosphate buffered saline (PBS) was flushed into the vagina and spread on a slide (SuperFrost™, Plus Adhesion Microscope Slides, Epredia). Slides were evaluated while wet, and the estrous cycle was determined by cell morphology as described by.^10^^,^^47^^,^^48^ When observed to be in the desired cycle stage, mice were euthanized as described below. H&E staining of the estrous cycle flushes was additionally done following conventional methods (Figure S1^47^). Stained slides were imaged with the Zeiss Axio Imager.M2m motorized light microscope with an EC PlanNeofluar 40x/0.75 objective, Zeiss Axiocam 506 color camera, and Zen 2.3 Pro software.

#### Tissue collection and processing

Mice were euthanized using carbon dioxide overdose followed by cervical dislocation. Uteri were dissected and fixed in ice-cold 4% PFA overnight. Tissues were then washed in 1× PBS and dehydrated stepwise to 70% ethanol and stored at +4°C until use.

The uterus was divided into single horns by cutting through the base of the uterus (cervix). The same horn was used for both paraffin sections and whole-mount immunostaining for comparable analysis. 2–3 mm pieces were cut from the base of the uterus (excluding the vagina) to be used for paraffin sections. For whole-mount immunostaining and SHG analysis, 4–5 mm pieces of uteri were used, coming from the middle of the uterine horns. Fixed uterine pieces for paraffin sections were dehydrated in increasing ethanol concentrations, washed in xylene, embedded in paraffin, and sectioned (5 μm) on microscope slides (SuperFrost™, Plus Adhesion Microscope Slides, Epredia). Slides were incubated at +37°C overnight.

#### Whole-mount immunohistochemistry

Whole-mount immunostaining was performed as follows (protocol adopted from^29^). Fixed mouse uterus tissues (described in Tissue collection and processing) were quenched overnight in freshly prepared absolute ethanol: DMSO: H_2_O_2_ (2:1:3, 30% H_2_O_2_ stock solution) at room temperature. Tissues were washed twice in absolute ethanol for 30 minutes per wash. Tissues then went through three freeze-thaw cycles, alternating tissue between -70°C and room temperature for at least an hour at each temperature. Tissues were rehydrated stepwise in TBST for 15 minutes at each step. Tissues were then blocked overnight in 10% serum (FBS and goat serum) in TBST. Following blocking, Tissues were incubated with FOXA2 (1:250 and 1:50, rabbit, Abcam ab108422) in blocking solution containing 5% DMSO for 48 hours. Following primary antibody incubation, the tissue was washed with TBST with 3 changes of solution, washing over 2 nights. Incubation in donkey anti-rabbit Alexa Fluor 594 (1:1000, Jackson ImmunoResearch 711-585-152) secondary antibody diluted in blocking solution containing 5% DMSO, filtered with a 0.45 μm filter syringe, was done for 48 hours. Samples were then washed with TBST for 3 nights, with 4 changes of solution. Tissue was then cleared with *Scale*CUBIC-1^45^ over 3 nights, changing *Scale*CUBIC-1 daily. All steps were done rotating at room temperature unless specified otherwise.

#### Microscopy

##### SHG imaging for paraffin-embedded slides

Estrous cycle slides were scanned with the Leica Stellaris 8 Dive multiphoton microscope with an HC FLUOTAR L 25x/0.95 W VISIR 0.17 water immersion objective. Scanning was done with slides still embedded in paraffin, as SHG is not affected by paraffin.^21^ A tunable 680–1300 nm dual output femtosecond laser (Coherent Chameleon Discovery) was set to 900 nm and used for excitation and the SHG signal was detected with a 4Tune HyD X detector, set to 445–455 nm in the epi direction. The scanning area was selected using the spiral function and the freeform drawing tool to select the entire uterine cross-section. Stitching was performed using the built-in Stitching program of the LAS X 4.5.0 software.

##### SHG and FOXA2 imaging for 3D uterine pieces

Whole-mount immunostained samples were imaged using a Nikon A1R MP+ multiphoton microscope with a tunable 680–1300 nm dual output femtosecond laser (Coherent Chameleon Discovery) and NIS-Elements C-ER acquisition software. All uteri pieces were imaged suspended in *Scale*CUBIC-1 in a custom-made chamber composed of two coverslips (24×32 mm #1 coverslip (Menzel-Gläser or Marienfeld) and 22×22 mm #1 coverslip (Menzel-Gläser) sealed with a ring of Blu-Tak (Bostik), described in.^21^ Uterine pieces were positioned laying down, with the lumen running parallel to the base of the chamber. Samples were scanned using a CF175 LWD 16×/0.8W DIC (WD 3.00 mm, Water) objective or a CF175 Apochromat 25× W MP1300 (NA 1.10, WD 2.0 mm, Water) objective. SHG and immunostaining signals were collected using both episcopic (epi) and transmitted (trans) direction non-descanned detectors, though only trans SHG and epi immunostaining signals were used for subsequent analysis. The excitation wavelength was set to 1080 nm for FOXA2 staining and 1100 nm for SHG estrous cycle samples, and transmitted signals were collected with a Nikon A1-NDN NA 1.2W condenser. In the epi direction, SHG was collected at 511–593 nm. In the trans direction SHG was collected at 495–560 nm. In the epi direction FOXA2 staining was collected above 593 nm long pass and above 560 nm long pass in the trans direction. A gradual, manually set, laser intensity compensation was used to maintain consistent signal intensity with increasing z-depth. All scans were acquired using 1 μm steps. For model training with FOXA2, *x×y* pixel sizes were 0.5 μm or 1.00 μm (×25), or 0.79 (×16) μm. For estrous cycle SHG analysis, *x×y* pixel sizes 0.79 μm. For 3D estrous cycle SHG analysis, all samples were scanned with the same laser settings, only laser compensation was changed between the samples due to variations in sample thickness and clearing.

#### Image processing and analysis

Stitching for scans acquired using the Leica Stellaris 8 Dive multiphoton was performed using the built-in Stitching program of the LAS X 4.5.0 software.

Image processing for SHG scans, FOXA2 immunostaining, and manual gland cage tracing was performed using Imaris software (Bitplane, Concord MA, USA; version 9.8.2). All files were converted to Imaris file format using the Imaris file converter (Version 9.8.2). Nikon A1R MP+ multiphoton scans were reconstructed in 3D automatically using the Imaris “Volume mode.”

##### FOXA2 immunostaining and surface rendering

Uterine gland-specific FOXA2 staining and the surrounding fibrillar collagen were simultaneously viewed by activating both Alexa Fluor 594 epi channels and the trans SHG channels. FOXA2 surface rendering was made using the “Surface” feature with the built-in algorithm. The Alexa Fluor 594 epi channel was used as the source channel. “Smooth” was activated, using 1.99 μm for surface detail. Absolute thresholding was used, and then a manual threshold value was set at 428.874. “Filter voxels” was set at above 10.0.

##### Gland cage manual tracing

To manually trace the gland cage outline, throughout the acquired z-stack, the “Surfaces” feature, the algorithm for manual editing was selected. Slices were annotated using the following sequence of tabs: Draw > Contour > Board. The “XY” orientation was used, with the following settings: resolution: auto, and visibility: none, so that each slice was annotated without seeing annotations from previous slices. The “Isoline” drawing mode was used. Manual tracing was done using the trans SHG channel, and annotations were made on every third slice. After annotations were completed, the surface was created and reconstructed in 3D and compared to the 3D surface rendering of the FOXA2 whole-mount staining by activating both surface rendering channels.

##### Isolation of individual glands from model

All SHG estrous cycle scans were segmented using the deep learning-based (semantic) segmentation model, and segmentations were viewed using 3D Slicer 5.6.2. To view segmentations as 3D models in Imaris, all segmentations were converted to a binary label map and exported as a 16-bit TIFF file, which were then converted to 8-bit TIFF files in Fiji (ImageJ). All 8-bit TIFF files were converted to Imaris files using the Imaris file converter (Version 9.8.2).

Once 3D segmentation label maps were opened in Imaris software, pixel dimensions were reset to match scanning parameters. To visualize single glands, a surface rendering was applied over the segmentation using the “Surface algorithm.” All 3D segmentation label maps, surface renderings were created using 1.95 μm smoothing for surface detail, absolute thresholding, and a manual threshold set at 62.4. No voxel filters were used. Once the surface was created, individual glands were isolated using the “Scissor” editing function.

##### 3D slice SHG intensity fold change images

To generate SHG intensity fold change images from the 3D scans in Figures 2E’–2H’, the image mean grey value was calculated in Fiji (ImageJ). Each image was divided by the mean grey value. A Rainbow RBG look up table was applied and scaled from 1 to 50.

##### Deep learning-based semantic segmentation model training

The primary objective was to develop a 3D semantic segmentation model capable of identifying the uterine gland “mold” in SHG scans. Using a supervised learning approach, the model required 3D ground truth masks, where each voxel is labeled as either 1 (uterine gland/lumen) or 0 (background). The computer model was trained using the following data sets: three scans with FOXA2 (ground truth for uterine glands) + SHG and five scans with SHG only (total of eight scans used for training). For data preparation, we divided the eight 3D scans into three distinct sets: four scans for training, two for validation, and two for testing. To improve efficiency, we selectively annotated a subset of 2D slices instead of the entire 3D scans. Specifically, 203 evenly distributed 2D slices (in the scanning orientation, every 15^th^ slice) were manually annotated across all eight 3D scans. Manual tracing was done using 3D Slicer 5.2.2. Tracing was done using the “Segmentation” mode with the “Scissors” tool with the following settings: for Operation, “fill inside” was selected, for Shape, “free-form” was selected, and for Slice Cut “Symmetric, 0.0000 mm” was selected. Using the annotated 2D slices and their corresponding masks, we initially trained a 2D semantic segmentation model based on the UNet architecture,^49^ with EfficientNet-B2 as its backbone.^50^ Once training was complete, the model was used to generate pseudo-labels for the remaining 2D slices without annotations. This process allowed us to construct complete 3D annotation masks for all scans. These fully annotated 3D scans were then utilized to train a 3D segmentation model, employing the UNet3D architecture^51^ to achieve the final goal. The run-time for the model depends on the computer hardware, especially the Graphical Processing Unit card. Our 1.5-minute run-time was based on the time the model took to segment a 3D scan using an Nvidia RTX 2080 ti graphics card.

### Quantification and statistical analysis

#### Intensity quantification

For paraffin section SHG intensity quantification: Only stitched uterine cross-section images (referred to as “merged” in LAS file names) were used for analysis, so the entire uterine cross-section could be analyzed.

All 2D paraffin-embedded SHG intensity measurements were performed in ImageJ (Fiji).^46^ Three tissue sections (each from a different mouse uteri)/estrous cycle stage were analyzed. SHG intensity measurements were taken using the “Measure” feature, collecting the following measurements: area, standard deviation, min & max gray value, and mean gray value.

To measure the endometrial stroma only in 2D paraffin-embedded tissue sections (Figure 1E), three measurements were collected from each sample’s endometrial stroma only, excluding glands, using a rectangular ROI placed in different stroma locations. The same ROI was used in all measurements and for all samples. The three collected measurements were averaged together and reported as an average SHG intensity per sample.

To measure the gland fibrillar collagen cage around gland SHG intensity (Figure 1F) and the fibrillar collagen cage/stroma ratio (Figure 1G), small, rectangular ROIs (approximately the width of the gland fibrillar collagen cage) or rectangular ROIs in the stroma, respectively, were used. For each estrous cycle sample, five measurements were taken from each of the following: gland fibrillar collagen cage and stroma. Gland fibrillar collagen cages were measured from five different uterine gland cross-sections. Stroma measurements were taken throughout the entire endometrium. The average of these measurements was used as a representative value for each category. Gland fibrillar collagen cage/stroma ratios were calculated by dividing the average gland fibrillar collagen cage mean intensity by the stroma mean intensity.

Fibrillar collagen cage thickness around glands (Figure 1H) was measured using Imaris software. All samples were analyzed with the following brightness settings: Min: 0, Max: 65, Gamma: 1. All measurements were done by opening the files in the “Slice” mode. Measurements were taken with the “Line” measurement type. Because the intensity and thickness of the SHG signal around the glands varied, a “Compass” approach was used, where measurements were made on the glands as if a compass had been laid on top of the gland, and the north, south, east, and west directions of the gland cages were measured. Measurements were made at these places, using clear boundaries between gland and stroma. 5 glands from each tissue section were measured, with glands selected throughout the tissue. Each “Compass” measurement was then averaged together for a representative measurement/gland. 5 gland averages were reported for each sample/estrous cycle stage.

3D SHG intensity quantification of the endometrial stroma (Figure 2I) was done using Imaris using the “Surface” program. To select the ROI used for SHG intensity quantification, the “Surface” algorithm was used with the option to segment a region of interest. Five 50x50x50 μm ROIs were placed throughout the endometrium, excluding the myometrium and lumen, and as much as possible, glands. All ROI placement was chosen using the “Slicer view”. The trans direction SHG channel was used as the source channel. “Smooth” was activated, using the automatically calculated 1.59 μm for surface detail. The “Absolute intensity” option was used for thresholding. The threshold was then set to 0, ensuring that the SHG intensity from the entire ROI cube was calculated. No additional filters were used. The SHG intensity was quantified using the “Statistics” feature, chosen from the “Detailed” tab, and “Specific values” selecting the “Intensity mean” value for the trans SHG channel.

To analyze SHG intensity fold change in fibrillar collagen cages in 3D samples (Figure 2J), all analysis was done in ImageJ (Fiji), and only the trans SHG channel was used for analysis. A sub-stack of the scan containing a gland cross-section cluster was manually selected and duplicated to use for analysis. A circular ROI was manually sized to fit around the gland cross-sections, and then the mean grey value was measured. The entire stack of images was divided by the mean grey value to determine the SHG intensity fold change of the gland fibrillar collagen cage. A 16-color look up table was then applied to the entire stack and set with a minimum of 1 to show the fold-change. A circular ROI was again placed over the gland cross-section cluster, and a histogram was used to analyze the mean value for the gland cluster for the entire stack.

#### Statistical analysis

The Shapiro–Wilk test of normality was used to confirm normal distribution of data along with visual evaluation of the distribution of data points. Homogeneity of variances was evaluated using Levene’s test. Comparisons between estrous cycle phases were performed using one-way ANOVA followed by Tukey post hoc test. All statistical tests were performed using GraphPad Prism 10.1.2, except for Levene’s test, which was performed using OriginPro 2024 software (OriginLab). All data are presented as individual data points and mean±s.d., and any time a data point represents the average of multiple measurements from within a sample is reported. The exact *P* values are shown on the graph when *P*≤0.05. For estrous cycle phase analysis, three mouse uteri per estrous cycle phase were collected and used for analysis (including scanning and reconstructions). For FOXA2 staining, one uterus, with three pieces stained independently, was used. For training the deep learning-based semantic segmentation model, scans from three different uteri were used.

### Additional resources

The code for the 2D and 3D uterine gland segmentation model is available at GitHub: https://github.com/imedslab/mice_uterine_gland_segmentation.

## References

[1] Garg A., Zielinska A.P., Yeung A.C., Abdelmalak R., Chen R., Hossain A., Israni A., Nelson S.M., Babwah A.V., Dhillo W.S. (2024). Luteal phase support in assisted reproductive technology. Nat. Rev. Endocrinol..

[2] Cooke P.S., Nanjappa M.K., Mesa A.M., Skinner M. (2018). Encyclopedia of Reproduction.

[3] DeLeon-Pennell K.Y., Barker T.H., Lindsey M.L. (2020). Fibroblasts: The arbiters of extracellular matrix remodeling. Matrix Biol..

[4] Naba A. (2024). Mechanisms of assembly and remodelling of the extracellular matrix. Nat. Rev. Mol. Cell Biol..

[5] Wei D., Su Y., Leung P.C.K., Li Y., Chen Z.J. (2024). Roles of bone morphogenetic proteins in endometrial remodeling during the human menstrual cycle and pregnancy. Hum. Reprod. Update.

[6] Gargett C.E., Chan R.W.S., Schwab K.E. (2008). Hormone and growth factor signaling in endometrial renewal: Role of stem/progenitor cells. Mol. Cell. Endocrinol..

[7] Coleman D.L., Kaliss N., Dagg C.P., Russell E.S., Fuller J.L., Staats J., Green M.C., Green E.L. (1966).

[8] Wood G.A., Fata J.E., Watson K.L.M., Khokha R. (2007). Circulating hormones and estrous stage predict cellular and stromal remodeling in murine uterus. Reproduction.

[9] Jin S. (2019). Bipotent stem cells support the cyclical regeneration of endometrial epithelium of the murine uterus. Proc. Natl. Acad. Sci. USA.

[10] Bertolin K., Murphy B.D., Croy B.A., Yamada A.T., DeMayo F.J., Adamson S.L. (2014). The Guide to Investigation of Mouse Pregnancy.

[11] Cooke P.S., Spencer T.E., Bartol F.F., Hayashi K. (2013). Uterine glands: Development, function and experimental model systems. Mol. Hum. Reprod..

[12] Kelleher A.M., Milano-Foster J., Behura S.K., Spencer T.E. (2018). Uterine glands coordinate on-time embryo implantation and impact endometrial decidualization for pregnancy success. Nat. Commun..

[13] Monsivais D., Nagashima T., Prunskaite-Hyyryläinen R., Nozawa K., Shimada K., Tang S., Hamor C., Agno J.E., Chen F., Masand R.P. (2021). Endometrial receptivity and implantation require uterine BMP signaling through an ACVR2A-SMAD1/SMAD5 axis. Nat. Commun..

[14] Rozario T., DeSimone D.W. (2010). The extracellular matrix in development and morphogenesis: A dynamic view. Dev. Biol..

[15] Tang M., Xu Y., Julian J., Carson D., Tabibzadeh S. (2005). Lefty is expressed in mouse endometrium in estrous cycle and peri-implantation period. Hum. Reprod..

[16] Gray C.A., Bartol F.F., Tarleton B.J., Wiley A.A., Johnson G.A., Bazer F.W., Spencer T.E. (2001). Developmental biology of uterine glands. Biol. Reprod..

[17] O’Connor B.B., Pope B.D., Peters M.M., Ris-Stalpers C., Parker K.K. (2020). The role of extracellular matrix in normal and pathological pregnancy: Future applications of microphysiological systems in reproductive medicine. Exp. Biol. Med..

[18] Ghajar C.M., Bissell M.J. (2008). Extracellular matrix control of mammary gland morphogenesis and tumorigenesis: insights from imaging. Histochem. Cell Biol..

[19] Tsutsui S., Wakasa H., Tsugami Y., Suzuki T., Nishimura T., Kobayashi K. (2020). Distinct Expression Patterns of Fibrillar Collagen Types I, III, and V in Association with Mammary Gland Remodeling during Pregnancy, Lactation and Weaning. J. Mammary Gland Biol. Neoplasia.

[20] Buchmann B., Engelbrecht L.K., Fernandez P., Hutterer F.P., Raich M.K., Scheel C.H., Bausch A.R. (2021). Mechanical plasticity of collagen directs branch elongation in human mammary gland organoids. Nat. Commun..

[21] Savolainen A., Kapiainen E., Ronkainen V.-P., Izzi V., Matzuk M.M., Monsivais D., Prunskaite-Hyyryläinen R. (2024). 3DMOUSEneST: a volumetric label-free imaging method evaluating embryo–uterine interaction and decidualization efficacy. Development.

[22] Aghigh A., Bancelin S., Rivard M., Pinsard M., Ibrahim H., Légaré F. (2023). Second harmonic generation microscopy: a powerful tool for bio-imaging. Biophys. Rev..

[23] Arora R., Fries A., Oelerich K., Marchuk K., Sabeur K., Giudice L.C., Laird D.J. (2016). Insights from imaging the implanting embryo and the uterine environment in three dimensions. Development.

[24] Besnard V., Wert S.E., Hull W.M., Whitsett J.A. (2004). Immunohistochemical localization of Foxa1 and Foxa2 in mouse embryos and adult tissues. Gene Expr. Patterns.

[25] Pálsson S., Cerri S., Poulsen H.S., Urup T., Law I., Van Leemput K. (2022). Predicting survival of glioblastoma from automatic whole-brain and tumor segmentation of MR images. Sci. Rep..

[26] Huynh N. (2023). Understanding Evaluation Metrics in Medical Image Segmentation. Medium.

[27] Müller D., Soto-Rey I., Kramer F. (2022). Towards a guideline for evaluation metrics in medical image segmentation. BMC Res. Notes.

[28] Taha A.A., Hanbury A. (2015). Metrics for evaluating 3D medical image segmentation: analysis, selection, and tool. BMC Med. Imaging.

[29] Alanentalo T., Asayesh A., Morrison H., Lorén C.E., Holmberg D., Sharpe J., Ahlgren U. (2007). Tomographic molecular imaging and 3D quantification within adult mouse organs. Nat. Methods.

[30] Granger K., Fitch S., Shen M., Lloyd J., Bhurke A., Hancock J., Ye X., Arora R. (2024). Murine uterine gland branching is necessary for gland function in implantation. Mol. Hum. Reprod..

[31] Teodoro W.R., Witzel S.S., Velosa A.P.P., Shimokomaki M., Abrahamsohn P.A., Zorn T.M.T. (2003). Increase of interstitial collagen in the mouse endometrium during decidualization. Connect. Tissue Res..

[32] Fata J.E., Werb Z., Bissell M.J. (2004). Regulation of mammary gland branching morphogenesis by the extracellular matrix and its remodeling enzymes. Breast Cancer Res..

[33] Khan S., Fitch S., Knox S., Arora R. (2022). Exocrine gland structure-function relationships. Development.

[34] Kelleher A.M., DeMayo F.J., Spencer T.E. (2019). Uterine Glands: Developmental Biology and Functional Roles in Pregnancy. Endocr. Rev..

[35] Vue Z., Gonzalez G., Stewart C.A., Mehra S., Behringer R.R. (2018). Volumetric imaging of the developing prepubertal mouse uterine epithelium using light sheet microscopy. Mol. Reprod. Dev..

[36] Inman J.L., Robertson C., Mott J.D., Bissell M.J. (2015). Mammary gland development: cell fate specification, stem cells and the microenvironment. Development.

[37] Brownfield D.G., Venugopalan G., Lo A., Mori H., Tanner K., Fletcher D.A., Bissell M.J. (2013). Patterned collagen fibers orient branching mammary epithelium through distinct signaling modules. Curr. Biol..

[38] Clause K.C., Barker T.H. (2013). Extracellular matrix signaling in morphogenesis and repair. Curr. Opin. Biotechnol..

[39] Rhodes J.M., Simons M. (2007). The extracellular matrix and blood vessel formation: not just a scaffold. J. Cell Mol. Med..

[40] Hsu J.C.f., Yamada K.M. (2010). Salivary gland branching morphogenesis- recent progress and future opportunities. Int. J. Oral Sci..

[41] Loganathan R., Little C.D., Rongish B.J. (2020). Extracellular matrix dynamics in tubulogenesis. Cell. Signal..

[42] Kučikas V., Werner M.P., Schmitz-Rode T., Louradour F., van Zandvoort M.A.M.J. (2023). Two-Photon Endoscopy: State of the Art and Perspectives. Mol. Imaging Biol..

[43] Zhang Y., Akins M.L., Murari K., Xi J., Li M.J., Luby-Phelps K., Mahendroo M., Li X. (2012). A compact fiber-optic SHG scanning endomicroscope and its application to visualize cervical remodeling during pregnancy. Proc. Natl. Acad. Sci. USA.

[44] Akins M.L., Luby-Phelps K., Mahendroo M. (2010). Second harmonic generation imaging as a potential tool for staging pregnancy and predicting preterm birth. J. Biomed. Opt..

[45] Susaki E.A., Tainaka K., Perrin D., Kishino F., Tawara T., Watanabe T.M., Yokoyama C., Onoe H., Eguchi M., Yamaguchi S. (2014). Whole-brain imaging with single-cell resolution using chemical cocktails and computational analysis. Cell.

[46] Schindelin J., Arganda-Carreras I., Frise E., Kaynig V., Longair M., Pietzsch T., Preibisch S., Rueden C., Saalfeld S., Schmid B. (2012). Fiji: An open-source platform for biological-image analysis. Nat. Methods.

[47] Byers S.L., Wiles M.V., Dunn S.L., Taft R.A. (2012). Mouse Estrous Cycle Identification Tool and Images. PLoS One.

[48] Caligioni C.S. (2009). Assessing reproductive status/stages in mice. Curr. Protoc. Neurosci..

[49] Ronneberger O., Fischer P., Brox T. (2015). Lecture Notes in Computer Science (including subseries Lecture Notes in Artificial Intelligence and Lecture Notes in Bioinformatics).

[50] Tan M., Le Q.V. (2019). Proceedings of the 36th International Conference on Machine Learning (PMLR 97).

[51] Kerfoot E., Clough J., Oksuz I., Lee J., King A.P., Schnabel J.A. (2019). Lecture Notes in Computer Science (including subseries Lecture Notes in Artificial Intelligence and Lecture Notes in Bioinformatics) 11395 LNCS.

