# Supplementary material for: Uterine collagen deposition fluctuates throughout the estrous cycle and provides a scaffold for gland visualization via the SHG-casting method
Source: iScience. 2025 Nov 10;28(12):113989. doi: 10.1016/j.isci.2025.113989 (PMC12704313; doi:10.1016/j.isci.2025.113989)
Supplement: Document S1. Figures S1 and S2 [file mmc1.pdf]

iScience, Volume *28*

Supplemental information

Uterine collagen deposition fluctuates throughout the estrous cycle and provides a scaffold for gland visualization via the *SHG-casting* method

Audrey Savolainen, Huy Hoang Nguyen, Egor Panfilov, Veli-Pekka Ronkainen, Aleksei Tiulpin, and Renata Prunskaite-Hyyryläinen

SUPPLEMENTARY FIGURE 1

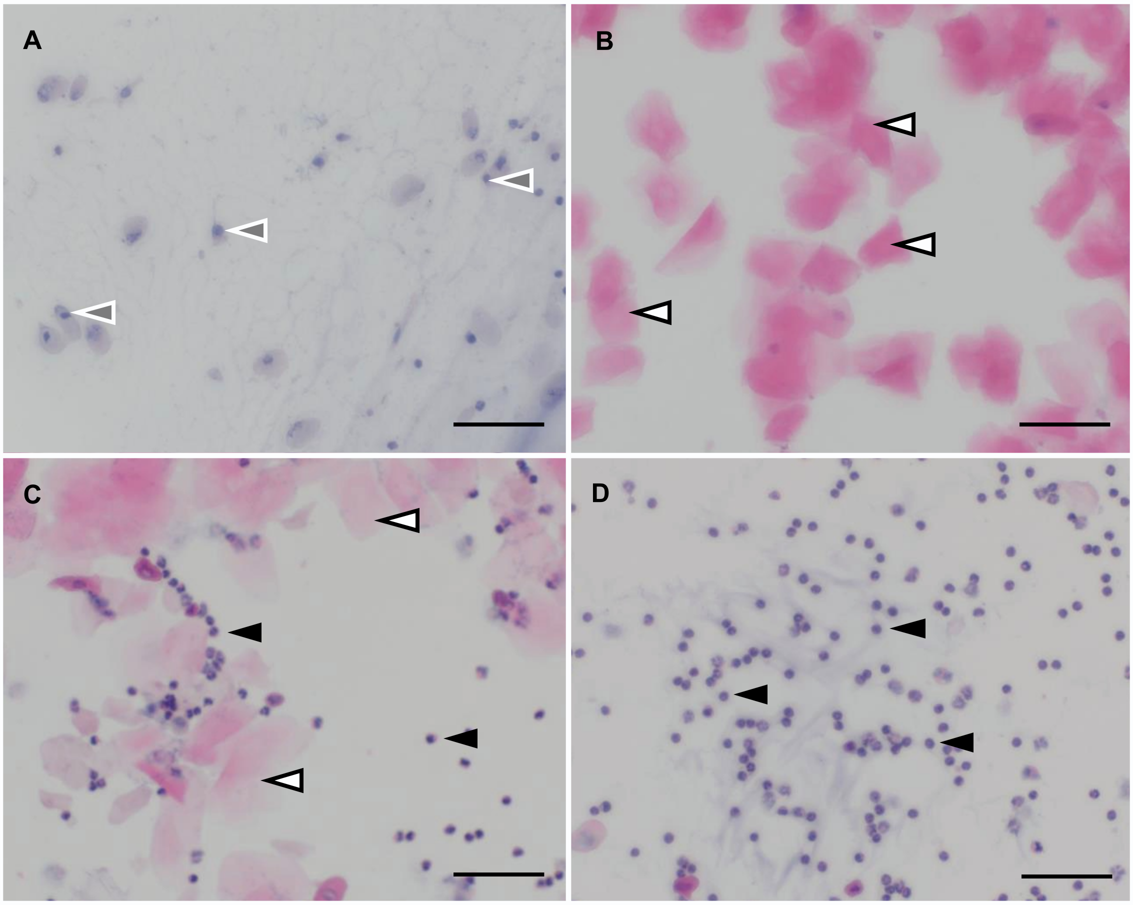

Supplementary Figure 1. **Mouse estrous cycle stage was determined by vaginal cytology.** Mouse uteri were collected at each stage of the estrus cycle. Representative vaginal cytology at each stage (**A**) nucleated epithelial cells (grey arrow) in proestrus, (**B**) cornified epithelial cells (white arrow) in estrus, (**C**) cornified epithelial cells (white arrow) and leukocytes (black arrow) in metestrus, (**D**) and leukocytes (black arrow) in diestrus. *n* = 3 uteri / estrous cycle stage. Scale bar is 50 µm.

SUPPLEMENTARY FIGURE 2

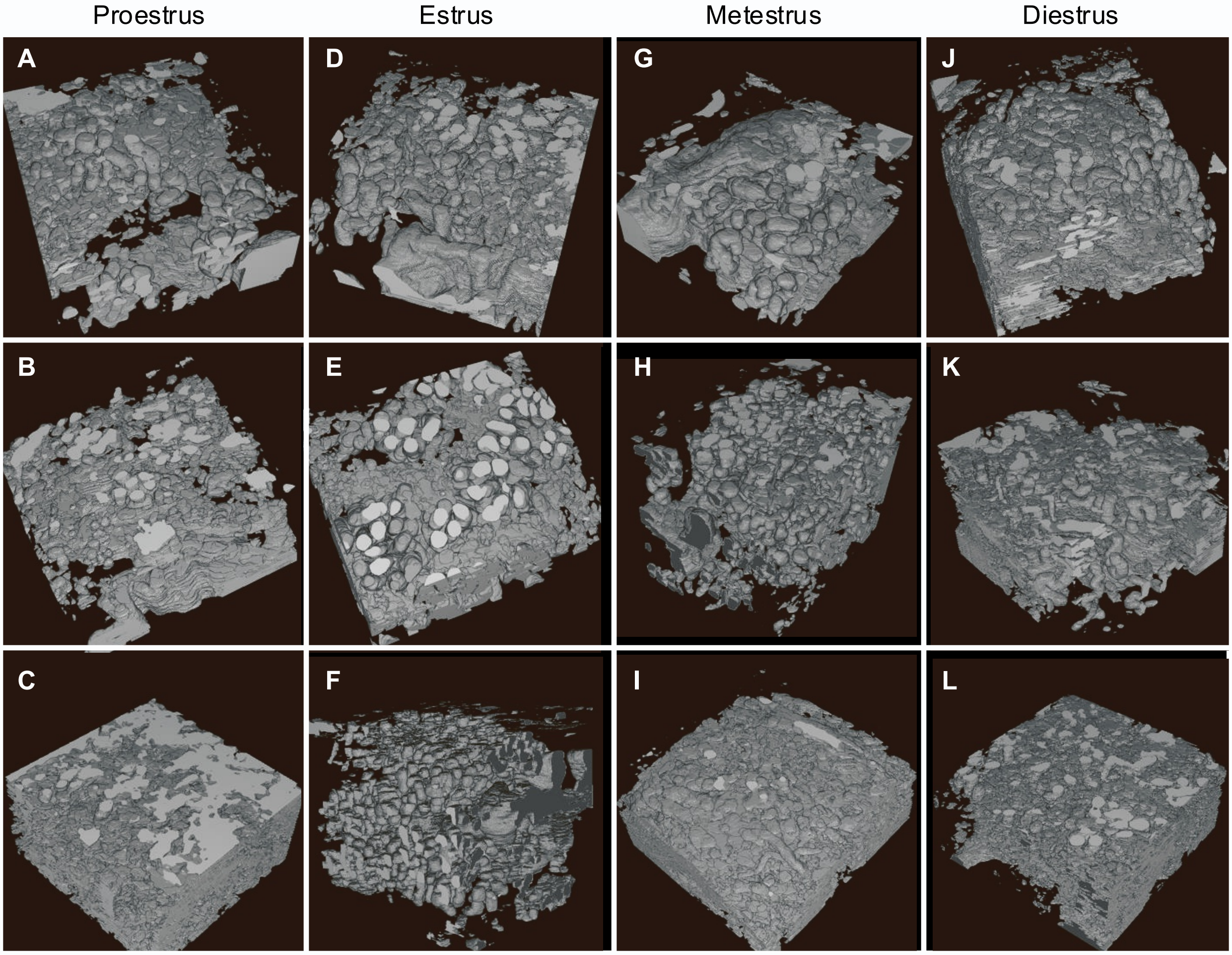

M

Proestrus		Estrus		Metestrus		Diestrus	
Scan (uteri) #	# of segmented glands	Scan (uteri) #	# of segmented glands	Scan (uteri) #	# of segmented glands	Scan (uteri) #	# of segmented glands
1	5	1	2	1	5	1	2
2	0	2	3	2	2	2	0
3	1	3	5	3	0	3	0
**Total**	**6**	**Total**	**10**	**Total**	**7**	**Total**	**2**

Supplementary Figure 2. **All *SHG-casting* segmentation model results from each estrous cycle.** SHG scans of each estrous cycle were used for SHG intensity quantification Figure 2, followed by segmentation using an automated *SHG-casting* segmentation model for gland morphology analysis. **A─C** shows segmentation results for proestrus scans. **D─F** shows segmentation results for estrus scans. **G─I** shows segmentation results for metestrus scans. **J─L** shows segmentation results for diestrus scans. The uterine glands derived from this data are shown in **Figure 8E─H**. **M)** Table depicting the number of glands segmented from individual uterine scans at various estrous cycle stages processed by the automated *SHG-casting* method. SHG; second harmonic generation. All segmentations come from separate scans of different uteri and have been cropped to exclude myometrium, emphasizing the endometrial glands. *n*=3 uteri / estrous cycle phase.
